# Intersubband Transition in GaN/InGaN Multiple Quantum Wells

**DOI:** 10.1038/srep11485

**Published:** 2015-06-19

**Authors:** G. Chen, X. Q. Wang, X. Rong, P. Wang, F. J. Xu, N. Tang, Z. X. Qin, Y. H. Chen, B. Shen

**Affiliations:** 1 State Key Laboratory of Artificial Microstructure and Mesoscopic Physics, School of Physics, Peking University, Beijing 100871, China; 2 Collaborative Innovation Center of Quantum Matter, Beijing, China; 3 Laboratory of Semiconductor Material Science, Institute of Semiconductors, CAS, Beijing 100083, China

## Abstract

Utilizing the growth temperature controlled epitaxy, high quality GaN/In_0.15_Ga_0.85_N multiple quantum wells designed for intersubband transition (ISBT) as novel candidates in III-nitride infrared device applications have been experimentally realized for the first time. Photo-absorption originated from the ISBT has been successfully observed at infrared regime covering the 3–5 μm atmosphere window, where the central absorption wavelength is modulated by adjusting the quantum well width. With increasing the quantum well thickness, the ISBT center wave length blue shifts at thickness less than 2.8 nm and then redshifts with further increase of the well thickness. The non-monotonic trend is most likely due to the polarization induced asymmetric shape of the quantum wells.

Since the pioneering discovery of intersubband transition (ISBT) in quantum structures, great concern has been aroused in this area, motivated by the tremendous prospect on infrared optoelectronics, such as quantum well infrared photodetectors (QWIPs), quantum cascade infrared photodetectors (QCIPs), quantum cascade lasers (QCLs), and electro-optic modulators^1^,^2^,^3^,^4^,^5^,^6^,^7^. Compared with traditional materials such as Si- or GaAs- based semiconductors, III-nitrides reap the benefit from their unique nature of large conduction band (CB) offset, ultra-short electron relaxation time and large LO-phonon energy^8^,^9^. Up to date, most efforts with this respect have been devoted to AlGaN/GaN multiple quantum wells (MQWs) towards the detection of 1.3 and 1.55 μm optical communication wavelengths as well as in the terahertz range^10^,^11^,^12^,^13^,^14^. However, due to the alloy disorder and defects scattering in the AlGaN barriers, it seems hard to acquire high-gain photoconductive response in the AlGaN/GaN MQWs. In addition, the lack of high electron mobility prevents such structures from achieving effective vertical transport across the MQWs. Those facts seriously limit the device performance and restrict the development of GaN-based ISBT detectors.

One of the possible solutions is to use GaN/InGaN MQWs instead. In this case, both of the alloy disorder and defects scattering in the barrier could be significantly reduced since the barrier is no longer AlGaN but GaN which exhibits much larger electron mobility^15^. Furthermore, the growth of GaN/InGaN MQW shave an advantage of easily avoiding cracks since the GaN/InGaN MQWs exhibit compressive strain^16^. However, it remains challenging in the preparation of InGaN upon how to precisely control the In fraction as well as the interface sharpness during the growth. And thus, only theoretical prediction of ISBT energy in GaN/InGaN MQWs have been reported so far^8^.

In this article, we will report an experimental observation of the inter-subband transition from c-plane GaN/In_0.15_Ga_0.85_N MQWs. Utilizing the growth temperature controlled epitaxy (GTCE) method in molecular beam epitaxy (MBE), high-quality GaN/In_0.15_Ga_0.85_N MQWs, which are nearly free of elemental inter-diffusion and phase separation, were successfully grown. The ISBT photo-absorption at infrared regime covering 3–5 μm atmosphere window was successfully achieved. With increasing the thickness of the quantum wells (*T*_*w*_), the central ISBT wavelength was found to show blue-shift in the MQWs at thickness no more than 2.8 nm and the red-shift with further increase of the well thickness.

## Results

**Figure 1:**
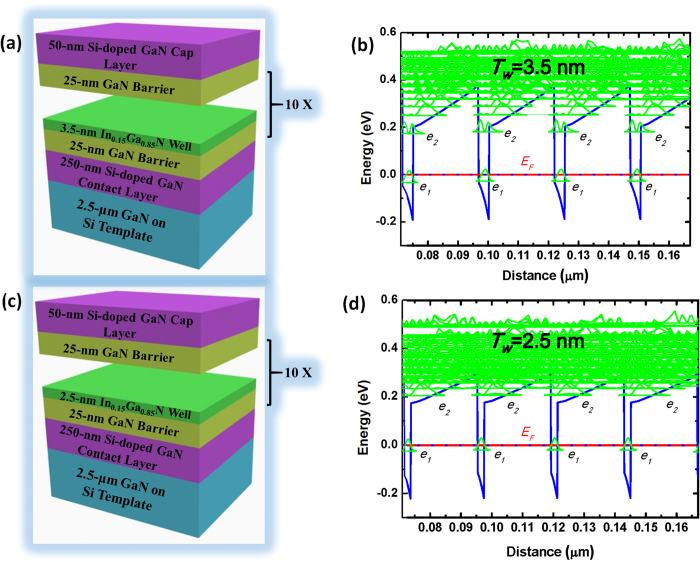
(**a**) Schematic image of structure and (**b**) conduction band profile of GaN/In_0.15_Ga_0.85_N (25 nm/3.5 nm) MQWs. (**c**) Schematic image of structure and (**d**) conduction band profile of GaN/In_0.15_Ga_0.85_N (25 nm/2.5 nm) MQWs.

We designed the structure of MQWs by self-consistently solving the Schrödinger and Poisson equations. The sample structures are shown in Fig. 1(a), and the corresponding band profiles are shown in Fig. 1(b), respectively. It is shown that the GaN barrier thickness (*T*_*b*_) is designed as 25 nm and the In composition is optimized to be 15% in InGaN wells for both types of quantum structure. It should be noted that the III-nitride materials exhibit strong internal electric field along c-direction^17^,^18^,^19^. That is referred as an intrinsic property of III-nitrides because of the asymmetric lattice structure. A c-plane GaN/InGaN MQW is asymmetric with a deep quantum well and a relatively wide triangular open mouth due to the polarization induced internal electric field^20^. Thus, the subbands in GaN-based MQWs structure can be divided into two types. One locates inside the well-confined quantum well and the other locates at the open mouth. In Fig. 1(a), the In_0.15_Ga_0.85_N quantum well thickness (*T*_*w*_) was set at 3.5 nm to make the first two subbands confined in the quantum well. In contrast, in Fig. 1(c), *T*_*w*_ was reduced to 2.5 nm to lift all the excited subbands to the open mouth of the quantum well. The latter structure is designed to allow a broad detection range from 200 meV (6.2 μm) to 550 meV (2.25 μm), which covers the range of the 3–5 μm atmosphere window. In both structures, the wells were heavily doped by Si to keep the ground subband below the Fermi level.

Samples were grown by plasma-assisted molecular beam epitaxy. A 2.5-μm-thick GaN layer grown on (111)Si substrate by metal-organic vapor phase epitaxy (MOVPE) was used as template. The (111)Si other than sapphire was used as substrate here is to avoid infrared absorption of the latter one. As shown in Fig. 1(a),(c), the growth started from a 250-nm-thick Si-doped n-type GaN layer, followed by a 25-nm-thick GaN barrier. Then 10 periods of GaN/In_0.15_Ga_0.85_N quantum wells were grown setting the barrier thickness of 25 nm. Finally, a 50-nm-thick n-type GaN cap layer was deposited. After optimization for the growth parameters, four samples with In_0.15_Ga_0.85_N well thicknesses of 2.5, 3, 3.5 and 4 nm were grown for comparison. For all the samples, Si was used as the n-type dopant in the InGaN quantum wells and the residual electron density was about 1 × 10^19^ cm^−3^.

**Figure 2:**
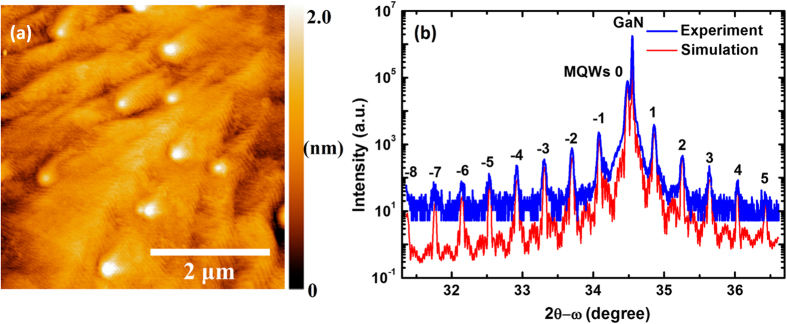
(**a**) Typical AFM image of surface morphology of the grown sample. (**b**) Experimental curve of HR-XRD (0002) 2θ-ω scans for the sample with *T*_*w*_ of 2.5 nm (blue line) and the corresponding simulated curve (red line).

As mentioned above, in order to grow high quality InGaN layers, the growth temperature controlled epitaxy is used here^21^, where the InGaN layer is grown at its maximum temperature of 620 °C at metal-rich condition, which leads to a flat surface and is helpful for improving the interface sharpness. Figure 2(a) shows the typical surface morphologies of the grown samples, which are free of cracks because the GaN/InGaN MQWs exhibit compressive strain when growing on the GaN:Si templates. The morphology is almost the same as that of homo-epitaxial GaN grown by MBE on MOVPE-grown GaN template. There are some spirals which are most likely due to the screw-component threading dislocations^22^. Except that, step-flow feature is clearly observed and the surface roughness (root mean square value) in a scanned area of 3 × 3 μm^2^ is less than 0.5 nm. This atomically flat surface coincides with the streaky pattern in the reflection high energy electron diffraction (RHEED) measurement. Figure 2(b) illustrates the high resolution x-ray diffraction (HR-XRD) 2θ-ω scans of the (0002) symmetric plane of the sample with *T*_*w*_ of 2.5 nm. The clearly visible satellite peaks from −8 to +5 orders indicate sharp interfaces between wells and barriers, and excellent periodicity of the MQWs. Estimated barrier and well thicknesses by simulation to the experimental XRD spectra are 23 and 2.3 nm, respectively, with the In composition in the InGaN quantum well of ~0.15, which coincide well with our theoretically designed values.

**Figure 3:**
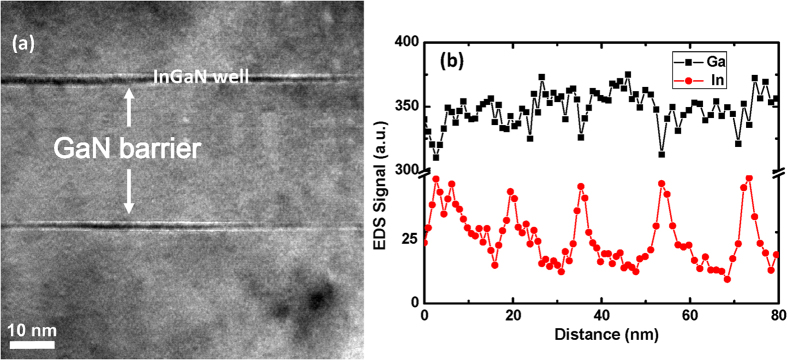
(**a**) Cross-sectional HR-TEM images of GaN/In_0.15_Ga_0.85_N MQWs. (**b**) Signal intensity of Ga (black) and In (red) atoms obtained by EDS. The distance is along the growth condition. The periodical distribution of In atom signal is clearly shown.

The interface sharpness of the MQWs is further revealed by high resolution transmission electron microscopy (HR-TEM) image shown in Fig. 3(a). Both the GaN barriers and In_0.15_Ga_0.85_N wells are uniform and the interfaces between them are edgily sharp. The composition uniformity of the alloy in the MQWs was investigated by space-resolved scanning of Ga and In atoms using energy dispersive spectrometry (EDS) measurement and their results are shown in Fig. 3(b). It is obvious that the scanned signal is of excellent periodicity and uniformity, being an evidence that the inter-diffusion and phase separation is effectively prevented by controlling the growth condition.

**Figure 4:**
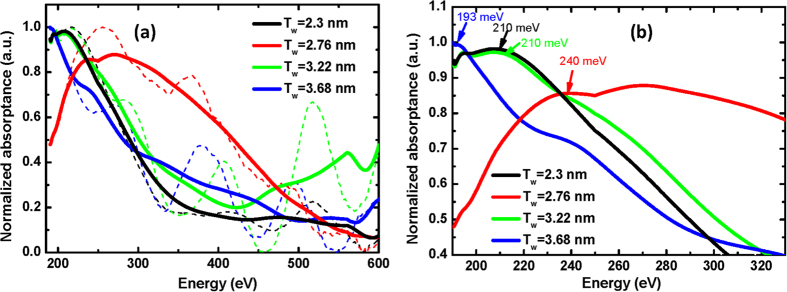
(**a**) Normalized absorption spectra (in dashed lines) and their corresponding fittings (in solid lines) of the GaN/In_0.15_Ga_0.85_N MQWs with different well thicknesses. (**b**) The zoomed in absorption spectra without interferenceof the GaN/In_0.15_Ga_0.85_N MQWs with different well thicknesses. The absorption peaks due to ISBT from *e*_*1*_ to *e*_*2*_are marked.

Figure 4 shows photoabsorption spectra measured by Fourier transform infrared spectrometer (FTIR). The 45-degree cut and polished sample waveguides were placed between the grid line polarizer and the detector. P-polarized light was selected as the signal while s-polarized one was set as background according to the optical transition selection rules^23^. The normalized intersubband absorption spectra are shown by dashed lines in Fig. 4(a), where periodic interference peaks caused by difference in refractive index between the epilayer and substrate are observed^24^. The fitted curves for the absorption spectra are also displayed in the figure. The ISBT-induced absorption peaks are visible in the range from 190 to 600 meV, in good agreement with the theoretical estimation. Figure 4(b) shows the zoomed in spectra which show the tenability of the ISBT-induced absorption peaks by adjusting the quantum well thickness. The peak positions of the absorption between the first two subbands (*E*_*12*_) can be estimated by Gaussian fits, which are 210, 240, 210 and 193 meV for *T*_*w*_  = 2.3, 2.8, 3.2, 3.7 nm (these thicknesses are estimated from the best fit to the XRD results), respectively. With increasing *T*_*w*_, the ISBT-induced absorption peak shows blueshift at *T*_*w*_ = 2.3 and 2.8 nm but redshift for the samples at *T*_*w*_ = 3.2 and 3.7 nm.

## Discussion

**Figure 5:**
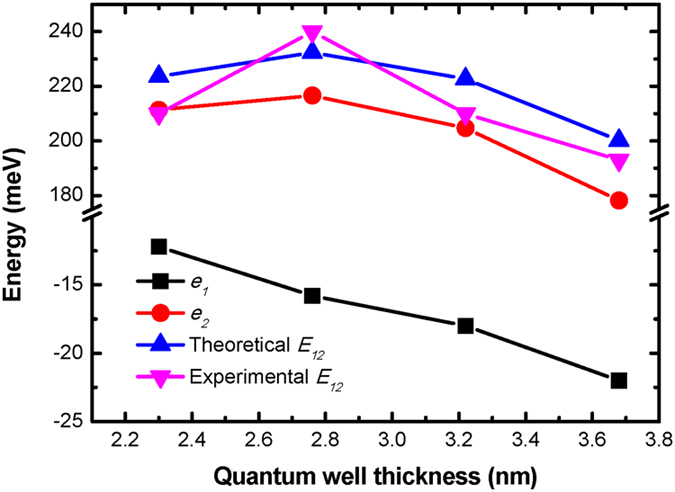
The energy level position of ground subband (*e*_*1*_, solid black squares), the second subband (*e*_*2*_, solid red circles), their theoretical interval energy (theoretical *E*_*12*_, solid pink triangles) and experiment interval energy (experimental *E*_*12*_, solid blue triangles) as a function of well thickness.

To figure out the reason why the two different structures show opposite tendency with increasing well thickness, theoretical simulation was performed. Photoabsorption oscillation strength in ISBT is known to depend on the wavefunction overlap of electrons in the ground state (*e*_*1*_) and the upper subbands. The component in the photo-absorption spectra aroused by *E*_*12*_ excitation plays a dominate role because their oscillation strength is much stronger than others^23^. Figure 5 shows calculated energy level position of the first two subbands (*e*_*1*_ and *e*_*2*_) and their interval energy (*E*_*12*_) as a function of the quantum well thickness. The experimental data are also included for comparison. The non-monotonic behavior of *E*_*12*_ is shown in the calculated results, which is in accord with the experiment data. It should be noted that this kind of non-monotonic dependence of intersubband interval energy on the quantum well thickness is similar to that of *E*_*13*_ but not *E*_*12*_in the GaN/AlN superlattices as reported by P. K. Kandaswamy *et al.*^25^. This non-monotonic behavior of *E*_*12*_ in the GaN/InGaN MQWs is attributed to that the *e*_*2*_position shifts from the open mouth of the well to the QW with increasing *T*_*w*_. In detail, when *T*_*w*_ is less than 2.8 nm, *e*_*1*_ is localized in the well and *e*_*2*_ locates at open mouth of the well. However, when *T*_*w*_ is larger than 2.8 nm, both *e*_*1*_ and *e*_*2*_ are in the wells. In the former case, the energy position of *e*_1_ decreases with increasing *T*_*w*_ since the electron quantum confinement gets weaker while the energy level of *e*_2_ was only slightly lifted with increasing *T*_*w*_ because the effective barrier height is increased^26^,^27^. Therefore, their interval energy (*E*_*12*_) gets larger. In the latter case, both *e*_*1*_ and *e*_*2*_ drop with increasing *T*_*w*_, but *E*_*12*_ becomes smaller since *e*_*2*_ falls quicker than *e*_*1.*_ Consequently, the photoabsorption peak blueshifts at first and then redshifts with increasing *T*_*w*_ from 2.3 to 3.7 nm. On the other hand, in GaN/AlN Superlattices, the conduction-band offset (CBO) (around 1.8 eV^25^) is large enough for the first two subbands (*e*_*1*_ and *e*_*2*_) confined in several-monolayer-thick GaN well while *e*_*3*_ locates around the open mouth of well. Thus *E*_*12*_ shows monotonic redshift and the *E*_*13*_shows non-monotonic behavior with the increasing well thickness, just as reported by P. K. Kandaswamy *et al.*^25^.

In summary, high quality GaN/In_0.15_Ga_0.85_N MQWs with ultra-sharp interfaces grown by MBE towards ISBT detectors in the atmospheric window have been investigated. The ISBT-induced photoabsorption at infrared region in the GaN/InGaN MQWs was successfully achieved, and the central wavelength can be effectively tuned from 5.2 to 6.4 μm by adjusting the thickness of the quantum wells. The central ISBT wavelength was first red shift and then blue shift in MQWs structure with increasing well thickness, which agrees with the theoretical estimation. The experimental evidence of ISBT in GaN/InGaN MQWs will open a new window for GaN based ISBT devices.

## Methods

### Fabrication

The samples were grown by plasma-assisted molecular beam epitaxy (SVTA). Atomic nitrogen was provided by an rf-plasma cell with typical nitrogen gas flow rate of 1.2 sccm. Ga, In and Si molecular beams were provided by conventional effusion cells with typical tell temperatures of 1000 °C, 860 °C and 1150 °C, respectively. The typical growth rate for the GaN and InGaN layer is about 1.5 Å/sec. The 2.5-μm-thick GaN layer, which were used as template for the quantum wells epitaxy, were grown on (111)Si substrate by low-pressure metal-organic vapor phase epitaxy (MOVPE) system by using trimethylgallium (TMG) and ammonia (NH_3_) as precursors, and H_2_/N_2_ as the carrier gas. Growth of the quantum wells was in-situ monitored by Reflection High-Energy Electron Diffraction (RHEED), where the streaky pattern was observed continuously, indicating an atomically flat surface.

### Numerial simulation

The self-consistent Schrödinger-Poisson equations assumed periodic potential conditions and charge neutrality conditions. In the calculation, the band gap was chosen to be 3.4 eV (0.65 eV) for GaN (InN), and the band gap bowing parameter for InGaN was set as 1.9 eV^21^. The ratio of conduction band offset to the total band offset was set as 65%. The lattice constants, spontaneous polarization and piezoelectric constants of GaN and InN were cited from Ref. 19. The thickness of InGaN quantum wells was carefully determined so that the intersubband transition (ISBT) energy between e_1_ and e_2_ is able to cover an infrared detection range of 3–5 μm atmosphere window.

### Measurements

The surface morphologies of the sample were analyzed by atomic force microscopy (AFM, Bruker Dimension Icon). The crystalline quality of the samples was characterized by high-resolution x-ray diffraction (HR-XRD, Bruker D8) and high-resolution transmission electron microscopy (HR-TEM, Tecnai F30). Residual carrier concentration was investigated by Hall-effect measurement system (ACCENT-HL5500). For infrared measurements, the sample was mechanically polished into 45° waveguides. The photoabsorption spectra were measured by Fourier transform infrared spectrometer (FTIR, Bruker IFS120).

## Additional Information

**How to cite this article**: Chen, G. *et al.* Intersubband Transition in GaN/InGaN Multiple Quantum Wells. *Sci. Rep.* **5**, 11485; doi: 10.1038/srep11485 (2015).
